# Changes in eating behaviors according to household income in adolescents during the COVID-19 pandemic: findings from the Korea National Health and Nutrition Examination Survey

**DOI:** 10.4178/epih.e2022102

**Published:** 2022-11-08

**Authors:** Hye Ah Lee, Ho Jung Lee, Bomi Park, Yoonhee Shin, Hyunjin Park, Hyesook Park

**Affiliations:** 1 Clinical Trial Center, Ewha Womans University Mokdong Hospital, Seoul, Korea; 2 Department of Preventive Medicine, Ewha Womans University College of Medicine, Seoul, Korea; 3 Department of Preventive Medicine, Chung-Ang University College of Medicine, Seoul, Korea; 4 Advanced Biomedical Research Institute, Ewha Womans University Seoul Hospital, Seoul, Korea; 5 College of Nursing, Ewha Womans University, Seoul, Korea; 6 Graduate Program in System Health Science and Engineering, Ewha Womans University, Seoul, Korea

**Keywords:** Adolescents, Feeding behavior, Socioeconomic factors, COVID-19

## Abstract

**OBJECTIVES:**

To assess social inequalities in changes in dietary behaviors among adolescents during the coronavirus disease 2019 (COVID-19) pandemic, we compared changes in dietary behavior indicators by household income.

**METHODS:**

Using cross-sectional data from the 2019 and 2020 Korea National Health and Nutrition Examination Survey, the prevalence of dietary behaviors in adolescents (12–18 years old) was estimated and changes in dietary behaviors during the COVID-19 pandemic were evaluated. We assessed changes in dietary behaviors with a household income (as a measure of socioeconomic status, SES) disparity.

**RESULTS:**

During the COVID-19 pandemic, the average consumption of vegetables decreased and food insecurity worsened. Adolescents were more likely to skip breakfast than before COVID-19 (33.1 and 37.4%). Soft drink consumption also increased in 2020 from 2019 (7.6 and 14.8%), especially among boys. Average sugar intake and sodium intake showed a tendency to decrease only in girls, but there was no significant difference according to SES level. Skipping breakfast was particularly evident in the low-SES group, and the difference according to household income level (high vs. low) was greater during COVID-19 than before. The prevalence of soft drink consumption increased significantly in the low-SES group, but the rate of increase did not differ by SES level.

**CONCLUSIONS:**

We found that the social disparity in skipping breakfast was further aggravated during the COVID-19 pandemic. To reach a better understanding of the dietary behaviors of adolescents, continuous monitoring is necessary.

## INTRODUCTION

Adolescence is a critical period involving psychosocial and physical changes, and proper nutrition for growth is important. In adolescence, individuals have more opportunities to choose food, and their food choices are greatly influenced by their peers and the environment, as well as their personal preferences. Furthermore, dietary behavior established during this period continues into adulthood, underscoring the importance of establishing healthy dietary behavior [1]. However, during the coronavirus disease 2019 (COVID-19) pandemic, changes in lifestyle have been detected as the time spent at home increased due to social distancing, school closures, and isolation due to illness or contact with confirmed cases. Increasingly many research papers have reported that adolescents’ lifestyle behaviors are also worsening [2]. According to a report from the United Nations International Children’s Emergency Fund, the COVID-19 crisis has made it difficult to access healthy food, as the consumption of vegetables and fruits is declining, while that of sugary drinks and snacks is increasing [3].

An individual’s dietary behavior is influenced by various determinants such as nutritional knowledge, dietary environments, and food availability. It is a well-known fact that there is a household income disparity in healthy food consumption. A report from the Prospective Urban Rural Epidemiology study in 18 countries demonstrated that the availability, affordability, and consumption of fruits and vegetables depended on household income [4]. Furthermore, previous studies reported that dietary behaviors such as skipping breakfast and soft drink consumption were also related to household income [5,6]. The COVID-19 pandemic has caused socioeconomic changes such as an increase in the number of unemployed and a contraction in economic activity [7,8]. A study conducted in San Francisco found that people facing new financial challenges due to the COVID-19 pandemic reported 10 times more sugary drink intake than those who did not [9]. Although some studies have evaluated changes in dietary behaviors and lifestyle during the COVID-19 pandemic, there has been insufficient research evaluating the behavioral changes of adolescents according to household income in the context of the COVID-19 pandemic.

Therefore, using data from a national survey of Koreans, we assessed the changes in dietary behaviors of adolescents (12–18 years old) during the COVID-19 pandemic and evaluated differences according to household income.

## MATERIALS AND METHODS

### Data

To assess changes in dietary behaviors during the COVID-19 pandemic, we analyzed data from the Korea National Health and Nutrition Examination Survey (KNHANES). The KNHANES is a survey representing the population of Korea that collects various data such as health status and nutritional status every year. Further information on KNHANES can be found in a previously published paper [10]. The latest data currently available are from the 2020 KNHANES. Therefore, we interpreted the data from 2019 as representing the period before COVID-19 and the data from 2020 as representing the period during the COVID-19 pandemic. The 2019 and 2020 surveys collected data from 8,110 and 7,359 people aged 1 year and over, and their response rates were 74.7% and 74.0%, respectively.

For this study, we included subjects aged 12–18 years (535 in 2019 and 454 in 2020). After excluding subjects who reported consuming <500 kcal/day or >5,000 kcal/day [11,12], 800 subjects were finally included (423 boys and 377 girls).

### Variables

As a measure of socioeconomic status (SES), we considered household income. Household income was expressed in quartiles (low, medium-low, medium-high, and high), which were defined based on the value of monthly household income divided by the square root of the household size.

In Korea, various indicators, including those related to nutrition, are set and monitored in the National Health Promotion Plan 2030. Taking into account the dietary behavior of adolescents along with these indicators, we considered 12 indicators of dietary behaviors; (1) family meal (yes), (2) nutrition education and counseling (yes), (3) use of nutrition labels (yes), (4) adequate fruit and vegetable intake (≥500 g/day of fruits and vegetables) [13,14], (5) adequate calcium intake (above the recommended intake of calcium, but below the upper limit), (6) adequate vitamin A intake (above the recommended intake of vitamin A, but below the upper limit), (7) skipping breakfast (≥5 day/wk), (8) excessive sodium intake (>2,300 mg/day of sodium intake) [11], (9) excessive fat intake (>30% of total energy intake [E%]), (10) excessive sugar intake (≥10 E%), (11) frequent soft drink consumption (≥5 times/wk), and (12) frequent eating out (≥once/day).

The definitions of indicators followed other studies or national monitoring standards. When it was inappropriate to estimate statistics, they were newly defined in consideration of the distribution of data. Family meal was defined as having breakfast or dinner with family. Nutrition education and counseling experience during the last year was defined based on a response of “yes” to that question. The use of nutrition labels was defined as those who responded that they checked the nutrition label when purchasing processed foods. In Korea, the National Health Promotion Plan recommends consuming more than 500 g of fruit and vegetable per day [13,14]. Data for daily fruit and vegetable intake were collected via 24-hour dietary recall survey. Daily nutrient intake was also estimated based on 24-hour dietary recall survey. The age-specific and sex-specific criteria for adequate intake of calcium and vitamin A were applied based on the 2020 Dietary Reference Intakes for Koreans [14]. According to the 2020 Dietary Reference Intakes for Koreans, a daily sodium intake of >2,300 mg was suggested as a required criterion for intervention to reduce the risk of chronic disease. Intakes of fat and sugar were calculated as a percentage of total energy intake (E%) to define indicators. Finally, the definition of eating out included take-out, food delivery, and school meals, and the frequency of eating out was classified into 2 groups according to whether participants reported eating out once or more per day.

### Statistical analysis

Considering the sampling method of the KNHANES, in summary statistics, we presented results as weighted means with 95% confidence intervals (CIs) for continuous data and unweighted frequencies with weighted percentages for categorical data. The results were produced by applying a sampling weight (i.e., wt_ntr) associated with the nutritional survey data.

We estimated the prevalence of basic characteristics and dietary-related indicators in adolescents before and during COVID-19. Differences in the prevalence of basic characteristics and dietary-related indicators before and during COVID-19 were evaluated using regression analysis. These differences were also analyzed by stratification by sex. For the 12 dietary behavior indicators, we also used regression analysis to estimate differences in the prevalence of dietary behaviors by high and low SES levels. The interaction effect of household income (high and low) and year (i.e., before and during COVID-19) on the prevalence of dietary behaviors was also assessed.

Statistical significance was assessed at p-value <0.05 under 2-tailed tests. All statistical analyses were performed using SAS version 9.4 (SAS Institute Inc., Cary, NC, USA).

### Ethics statement

There was no personal information in the open KNHANES data and ethical approval for the use of open KNHANES data was exempted from the Institutional Review Board Committee of the Ewha Womans University Hospital (IRB No. EUMC 2022-05-028).

## RESULTS

Table 1 shows changes in dietary behaviors during COVID-19. Regarding healthy dietary behavior, such as family meals and adequate fruit and vegetable intake, there were no significant changes, but the average daily vegetable intake showed a tendency to decrease (170.7 g/day in 2019 and 149.8 g/day in 2020; p=0.07). The prevalence of skipping breakfast in 2020 was 37.4%, an increase of about 4.3%p compared to 2019, but the change was not significant. Meanwhile, the prevalence of frequent soft drink consumption (≥5 times/wk) increased significantly from 7.6% to 14.8% (p<0.01). This change was evident in boys, not in girls. The prevalence of frequent eating out (≥once/day) decreased from 46.0% to 36.8% (p<0.05). In terms of improved health behavior, only in girls, the average sugar intake showed a tendency to decrease, and excessive sodium intake (>2,300 mg/day of sodium intake) was reduced by 12.7%p. Additionally, the number of people belonging to the low or medium-low level of food security increased compared to pre-COVID-19.

When assessed according to household income, the prevalence of using nutrition labels and excessive sodium intake differed during the COVID-19 pandemic. The prevalence of using nutrition labels was higher in participants with a low SES level and lower in those with a medium-high SES level. Excessive sodium intake was higher in participants with a medium-high SES level and lower in those with a medium-low SES level. Compared to the high SES level, although statistical significance was not met, the prevalence of family meals (Δ high-low=11.5%), adequate calcium intake (Δ high-low=4.9%), and eating out (Δ high-low= 21.1%) in participants with low SES levels during COVID-19 was lower. Furthermore, the social disparity in breakfast skipping was further aggravated, which was found to be significant (Table 2).

**Figure 1:**
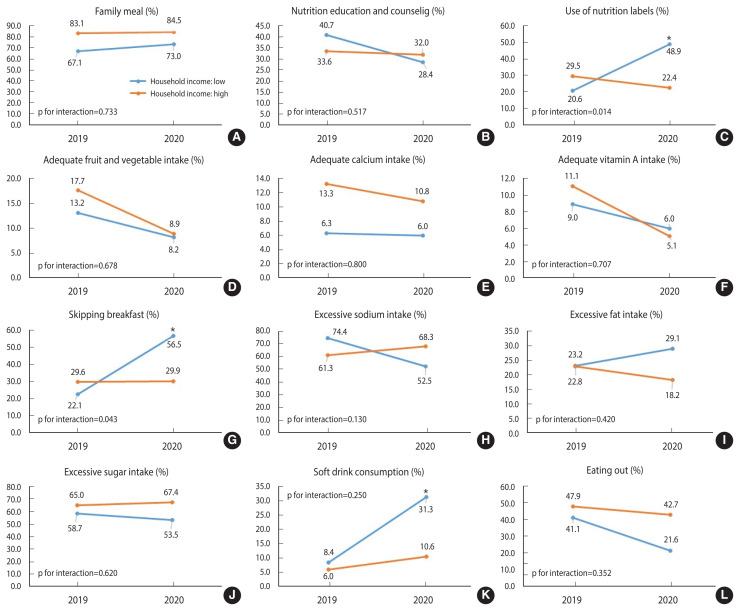
Changes in dietary behaviors by high and low household income in adolescents (12–18 years old) before and during COVID-19 (2019 vs. 2020); (A) family meal, (B) nutrition education and counseling, (C) use of nutrition labels, (D) adequate fruit and vegetable intake, (E) adequate calcium intake, (F) adequate vitamin A intake, (G) skipping breakfast, (H) excessive sodium intake, (I) excessive fat intake, (J) excessive sugar intake, (K) soft drink consumption, and (L) eating out. Results are expressed as a weighted percentage (%) taking into account the sampling method. COVID-19, coronavirus disease 2019. *p<0.05, it represents a statistically significant change before and during COVID-19 within that socioeconomic status group.

The changes in dietary behaviors during COVID-19 among adolescents with low and high SES are presented in Figure 1. The prevalence of skipping breakfast among those with a low SES level increased significantly compared to before COVID-19 (22.1% in 2019→56.5% in 2020). This increase was much greater than was observed among adolescents with a high SES level (29.6% in 2019 →29.9% in 2020; p<0.05). Soft drink consumption increased in both high-SES and low-SES groups compared to before COVID-19, and the increase in the low-SES group was larger than that in the high-SES group (low SES: 22.9%p vs. high SES: 4.6%p), but it did not reach a statistically significant level. Meanwhile, the prevalence of adequate fruit and vegetable intake tended to decrease at both high and low SES levels. The prevalence of adequate vitamin A intake also declined in both SES groups compared to before COVID-19. Even when estimating the prevalence of dietary behaviors from 2016, the social disparity in skipping breakfast, family meals, and adequate calcium intake during the COVID-19 period was found to have deepened (Supplementary Material 1).

## DISCUSSION

In our study, from national data, we evaluated changes in dietary behaviors of adolescents during COVID-19. Overall, soft drink consumption increased by about 7.2%p, and this change was evident in boys, not in girls (9.5%p in boys and 4.4%p in girls). Although no significant change was observed in the entire study group, the average vegetable intake and calcium intake significantly decreased in girls. During the COVID-19 pandemic, the increase in breakfast skipping was more pronounced in adolescents with low SES, and the change was significantly greater than in those in adolescents with high SES (low SES: 34.4%p vs. high SES: 0.3%p). Soft drink consumption also increased significantly in participants with a low SES level (8.4% in 2019→31.3% in 2020), but the difference was not significant when compared to changes in participants with a high SES level (4.6%p).

In March 2020, the Ministry of Education of the Korea postponed the start of school, middle and high school students started online school in early April, and face-to-face classes sequentially started in May when COVID-19 was stagnant. However, in August, as social distancing was upgraded to level 2 for early mitigation of the second wave of COVID-19, online classes were expanded [15]. The decline in the prevalence of eating out during COVID-19 may have been attributed to social distancing and school closures. The unprecedented COVID-19 pandemic has affected many aspects of daily life, and behavioral changes during the COVID-19 pandemic have been detected in many studies [3,9,16,17]. Due to fear of COVID-19 infection, changes have also been made in the way food is purchased. Food and groceries have been purchased through delivery systems, and purchases of frozen or canned food, which have a longer shelf life than fresh food, have increased [18]. This likely contributed to the decrease in fruit and vegetable intake. A study conducted in 3 European countries observed a decrease in average consumption of fruits and vegetables and an increase in ready-made meals during the lockdown of the COVID-19 pandemic [19]. Interestingly, some studies have shown a trend toward an increase in healthy dietary behaviors. Studies conducted in Italy [20], Spain [21], and Czech Republic [22] during the COVID-19 pandemic reported increased consumption of fruits and vegetables. As an explanation for this, adolescents’ food choices may have been affected because they were confined to the home and controlled under parental sanctions. Consistent with previous research [16], an increase in soft drink consumption among adolescents was clearly observed during COVID-19 in our study. However, this has also shown mixed results between studies [20,23].

In our study, there was a significant increase in breakfast skipping and soft drink consumption among adolescents with low SES. Although changes in household income have not been captured, the proportion of adolescents with low or medium-low levels of food security tended to increase during COVID-19 (Table 1). Exposure to food insecurity in adolescence can cause extreme psychological stress and affect the mental health and well-being of adolescents [24]. It has also been reported that food insecurity is associated with poor dietary behavior and that access to healthy food may be restricted [25]. As one of the major determinants of food insecurity, low household income levels can be considered [26]. SES is known to be a determinant of individuals’ dietary behavior [27]. A study conducted mainly in Denmark, Germany, and Slovenia reported that the loss of household income due to the pandemic was related to changes in food consumption patterns [19]. Those with low SES are more likely to be exposed to vulnerable environments, which is also associated with access to and consumption of unhealthy foods [28]. A study in Korean adolescents reported that adolescents with lower scores on the family affluence scale were more likely to skip breakfast and frequently consume soda, fast food, and instant noodles [29]. A study in high-school subjects in Spain observed that the risk of the diet worsening was 21% higher in adolescents with more disadvantaged SES [30]. Barriers to high-priced healthy food increase the choice of affordable and accessible energy-dense or nutrient-poor foods [31,32]. The government-funded meal support program for low-income children and adolescents did not guarantee continuous support as schools were closed due to social distancing during the COVID-19 period. As a result of this, it is likely that the nutritional status of children and adolescents with low SES was affected. In this context, among adolescents with low SES, there was a significant increase in breakfast skipping, along with a decrease in adequate fruit and vegetable intake. In order to close the gap according to the SES level, it seems that continuing national-level support is needed.

There are some points to keep in mind when interpreting the results. This study did not assess changes in dietary behavior within individuals. Thus, while our study may show changes in overall status, we have not been able to directly assess the causes of changes in dietary behaviors. Nevertheless, our findings provide useful information to understand the dietary behaviors of adolescents in the environment of the COVID-19 pandemic. In addition, from the perspective of the life-course, focusing on adolescence, which is closely related to the health and behavior of adults, we evaluated differences according to SES levels, as well as differences between before and during COVID-19. Although the analysis was performed considering the multi-stage sampling method, there may have been problems with the validity of estimates due to the small sample size in subcategories. Therefore, we did not assess social disparities for changes in dietary behavior by sex.

In Korea, social distancing was lifted on April 18, 2022. The question raised after the easing of social distancing concerns whether these trends will continue. Therefore, it will be necessary to continue monitoring these changes and to check whether dietary patterns are maintained in the generation that experienced COVID-19 in adolescence. Obesity among children and adolescents in Korea increased significantly during COVID-19 [33], and we observed some deterioration in health behaviors among adolescents in our study. Therefore, it is necessary to reduce obesity and to intervene before poor health behavior becomes a habit.

## Figures and Tables

**Table 1.** Basic characteristics and changes in dietary behaviors of adolescents (12-18 years old) before and during COVID-19

Characteristics		Total			Boys			Girls		
		2019 (before COVID-19)	2020 (during COVID-19)	p-value for 2020 vs. 2019	2019 (before COVID-19)	2020 (during COVID-19)	p-value for 2020 vs. 2019	2019 (before COVID-19)	2020 (during COVID-19)	p-value for 2020 vs. 2019
Sex (male)		233 (52.3)	190 (55.3)	0.457	-	-		-	-	
Age (yr)		15.3 (15.07, 15.43)	15.1 (14.85, 15.39)	0.437	15.3 (15.06, 15.59)	15.3 (14.88, 15.68)	0.854	15.2 (14.91, 15.43)	14.9 (14.62, 15.24)	0.251
Household income										
	Low	49 (12.5)	23 (6.8)	0.132	25 (11.8)	11 (4.9)	0.018	24 (13.2)	12 (9.1)	0.427
	Medium-low	129 (27.4)	89 (24.1)		66 (28.5)	40 (18.8)		63 (26.2)	49 (30.6)	
	Medium-high	133 (31.5)	132 (39.2)		69 (31.3)	75 (40.1)		64 (31.7)	57 (38.0)	
	High	145 (28.7)	98 (30.0)		72 (28.4)	63 (36.2)		73 (28.9)	35 (22.3)	
Food security										
	Low	0 (0.0)	1 (0.3)	0.036	0 (0.0)	0 (0.0)	0.017	0 (0.0)	1 (0.6)	0.468
	Medium-low	5 (1.2)	9 (3.5)		1 (0.6)	5 (3.1)		4 (1.9)	4 (3.8)	
	Medium-high	164 (38.8)	105 (29.6)		90 (42.1)	58 (28.3)		74 (35.2)	47 (31.3)	
	High	288 (60.0)	228 (66.7)		142 (57.3)	127 (68.6)		146 (62.9)	101 (64.3)	
Nutrition and food intake										
	Total energy (kcal)	2,039.7 (1,951.6, 2,127.8)	1,955.1 (1,866.6, 2,043.7)	0.173	2,237.2 (2,104.5, 2,369.9)	2,186 (2,048.3, 2,323.7)	0.600	1,823.3 (1,712.4, 1,934.3)	1,669.5 (1,559.4, 1,779.6)	0.051
	Average daily fruit intake (g)	88.5 (72.2, 104.9)	89.8 (48.7, 130.9)	0.952	80.3 (58.4, 102.2)	97.7 (29.9, 165.4)	0.628	97.5 (76.1, 118.8)	80.1 (56.9, 103.4)	0.280
	Average daily vegetable intake (g)	170.7 (153.9, 188.4)	149.8 (135.0, 165.4)	0.071	192 (165.5, 220.5)	178.5 (157.0, 201.3)	0.443	148.8 (129.8, 169.2)	117.8 (97.3, 140.2)	0.038
	Average daily calcium intake (mg)	422.1 (395.5, 450.53)	394.4 (362.7, 428.92)	0.202	449.4 (403.6, 500.1)	448.8 (398.9, 504.9)	0.987	394.1 (363.6, 427.2)	336.2 (305.6, 369.8)	0.012
	Average daily vitamin A intake (μgRAE)	272.2 (246.0, 301.2)	264.9 (238.4, 294.5)	0.712	311.5 (273.1, 355.5)	297.5 (257.9, 343.2)	0.635	234.8 (208.5, 264.5)	229.5 (198.8, 265.0)	0.811
	Average daily sodium intake (mg)	2,799.4 (2,635.8, 2,973.2)	2,672.7 (2,482.4, 2877.5)	0.335	3,162.6 (2,888.1, 3,463.3)	3,182.4 (2,917.0, 3,472.0)	0.922	2,449.2 (2,266.2, 2,647.1)	2,153.5 (1,942.2, 2,387.7)	0.054
	Average daily fat intake (E%)	25.1 (24.1, 26.0)	25.5 (24.4, 26.6)	0.574	25.1 (23.7, 26.4)	25.7 (24.1, 27.2)	0.577	25.1 (23.9, 26.3)	25.3 (23.7, 26.8)	0.838
	Average daily sugar intake (g)	54.7 (50.0, 59.7)	50.4 (45.9, 55.3)	0.202	53.5 (46.6, 61.5)	52.7 (46.6, 59.6)	0.866	55.9 (49.8, 62.8)	47.6 (41.8, 54.2)	0.065
Indicators of eating behavior										
	Family meals	358 (73.7)	277 (79.7)	0.075	186 (74.3)	156 (81.4)	0.143	172 (73.2)	121 (77.6)	0.365
	Nutrition education and counseling	150 (30.5)	109 (27.3)	0.420	71 (27.8)	58 (25.0)	0.580	79 (33.5)	51 (30.1)	0.576
	Use of nutrition labels	116 (26.7)	87 (25.6)	0.770	47 (21.0)	41 (22.6)	0.730	69 (32.9)	46 (29.3)	0.522
	Adequate fruit and vegetable intake (≥ 500 g/day of fruits and vegetables)	69 (13.8)	38 (12.6)	0.718	42 (14.8)	25 (14.5)	0.950	27 (12.6)	13 (10.3)	0.548
	Adequate calcium intake (above the recommended intake of calcium, but below the upper limit)	42 (8.0)	31 (9.2)	0.593	27 (8.6)	24 (13.8)	0.118	15 (7.4)	7 (3.5)	0.119
	Adequate vitamin A intake (above the recommended intake of vitamin A, but below the upper limit)	44 (9.3)	25 (7.3)	0.438	24 (9.3)	16 (8.2)	0.734	20 (9.3)	9 (6.1)	0.371
	Skipping breakfast (≥5 day/wk)	147 (33.1)	130 (37.4)	0.309	77 (33.0)	68 (35.6)	0.669	70 (33.2)	62 (39.6)	0.259
	Excessive sodium intake (>2,300 mg/day of sodium intake)	316 (66.3)	218 (64.3)	0.624	186 (74.2)	149 (80.0)	0.258	130 (57.6)	69 (44.9)	0.028
	Excessive fat intake (>30 E%)	113 (24.4)	96 (27.2)	0.449	56 (22.6)	50 (25.1)	0.616	57 (26.4)	46 (29.8)	0.534
	Excessive sugar intake (≥10 E%)	300 (63.6)	210 (62.4)	0.759	142 (57.1)	110 (57.1)	0.997	158 (70.7)	100 (68.9)	0.741
	Soft drink consumption (≥5 times/wk)	36 (7.6)	52 (14.8)	0.002	19 (7.7)	34 (17.2)	0.005	17 (7.5)	18 (11.9)	0.206
	Eating out (≥1/day)	195 (46.0)	124 (36.8)	0.038	91 (45.5)	83 (43.7)	0.788	104 (46.5)	41 (28.1)	0.001

Values are presented as weighted mean (95% confidence interval) for numerical data and unweighted frequency (weighted %) for categorical data. COVID-19, coronavirus 2019; RAE, retinol activity equivalents.

**Table 2.** Changes in dietary behaviors by household income levels in adolescents (12-18 years old) before and during COVID-19 (2019 vs. 2020)

Variables	Household income				High-Low (Δ), %	p for High-Low (Δ)	p for the DID
	Low	Medium-low	Medium-high	High			
Family meal							0.733
2019	33 (67.1)	101 (77.4)	100 (64.0)	123 (83.1)	16.0	0.098	
2020	16 (73.0)	67 (74.5)	110 (80.6)	84 (84.5)	11.5	0.219	
Nutrition education and counseling							0.517
2019	18 (40.7)	43 (29.0)	43 (25.7)	46 (33.6)	-7.1	0.528	
2020	8 (28.4)	28 (27.1)	39 (23.6)	34 (32.0)	3.6	0.762	
Use of nutrition labels							0.014
2019	9 (20.6)	28 (19.6)	38 (33.3)	41 (29.5)	8.8	0.315	
2020	10 (48.9)	31 (33.8)	28 (18.6)	17 (22.4)	-26.6	0.017	
Adequate fruit and vegetable intake (≥500 g/day of fruits and vegetables)							0.678
2019	8 (13.2)	17 (12.2)	17 (12.1)	27 (17.7)	4.5	0.517	
2020	3 (8.2)	8 (9.8)	20 (17.7)	6 (8.9)	0.7	0.907	
Adequate calcium intake (above the recommended intake of calcium, but below the upper limit)							0.800
2019	2 (6.3)	8 (4.5)	10 (7.2)	22 (13.3)	7.0	0.163	
2020	1 (6.0)	5 (6.7)	13 (10.1)	12 (10.8)	4.9	0.483	
Adequate vitamin A intake (above the recommended intake of vitamin A, but below the upper limit)							0.707
2019	5 (9.0)	10 (7.5)	11 (9.6)	18 (11.1)	2.2	0.676	
2020	1 (6.0)	6 (8.2)	13 (8.6)	5 (5.1)	-0.9	0.888	
Skipping breakfast (≥5 day/wk)							0.043
2019	14 (22.1)	52 (39.8)	36 (33.1)	44 (29.6)	7.4	0.358	
2020	13 (56.5)	35 (36.8)	51 (40.4)	31 (29.9)	-26.6	0.073	
Excessive sodium intake (>2,300 mg/day of sodium intake)							0.130
2019	39 (74.4)	95 (69.6)	88 (61.3)	88 (61.3)	-13.1	0.285	
2020	14 (52.5)	49 (51.2)	90 (71.3)	64 (68.3)	15.8	0.271	
Excessive fat intake (>30 E%)							0.420
2019	11 (23.2)	35 (26.3)	30 (25.3)	37 (22.8)	-0.4	0.958	
2020	5 (29.1)	29 (29.9)	42 (32.3)	20 (18.2)	-10.9	0.336	
Excessive sugar intake (≥10 E%)							0.620
2019	29 (58.7)	78 (57.9)	92 (68.3)	100 (65.0)	6.3	0.490	
2020	12 (53.5)	52 (63.5)	77 (59.6)	69 (67.4)	13.9	0.254	
Soft drink consumption (≥5 times/wk)							0.250
2019	5 (8.4)	14 (11.9)	7 (5.2)	10 (6.0)	-2.4	0.580	
2020	5 (31.3)	12 (13.9)	23 (15.9)	12 (10.6)	-20.8	0.179	
Eating out (≥1/day)							0.352
2019	16 (41.1)	53 (41.7)	55 (48.5)	70 (47.9)	6.8	0.535	
2020	8 (21.6)	31 (34.6)	46 (36.3)	39 (42.7)	21.1	0.056	

Values are presented as unweighted frequency (weighted %) taking into account the sampling method. COVID-19, coronavirus disease 2019; DID, difference in difference.
