# Supplementary material for: Changes in eating behaviors according to household income in adolescents during the COVID-19 pandemic: findings from the Korea National Health and Nutrition Examination Survey
Source: Epidemiol Health. 2022 Nov 8;44:e2022102. doi: 10.4178/epih.e2022102 (PMC10185980; doi:10.4178/epih.e2022102)

Supplementary Material 1. Changes in eating behaviors by high and low household income in adolescents (12-18 years old) from 2016 to 2020.


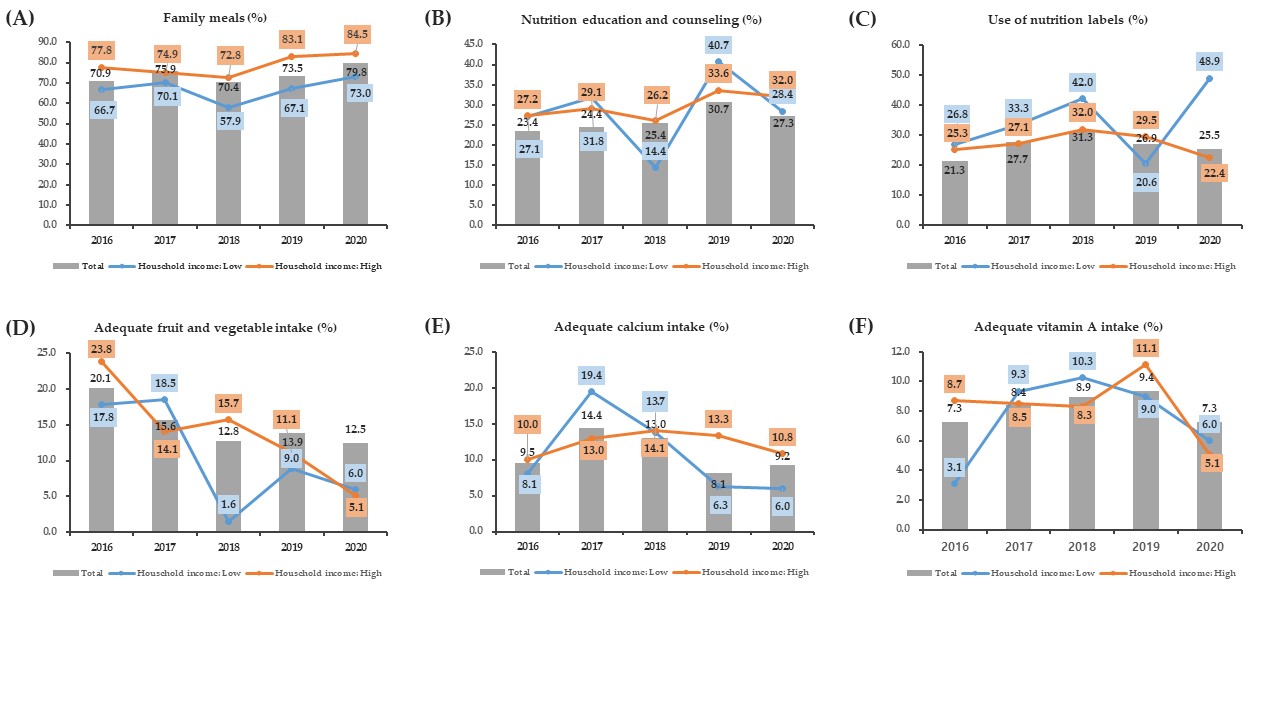


Results are expressed as a weighted percentage (%) taking into account the sampling method.

Supplementary Material l. (Continued).


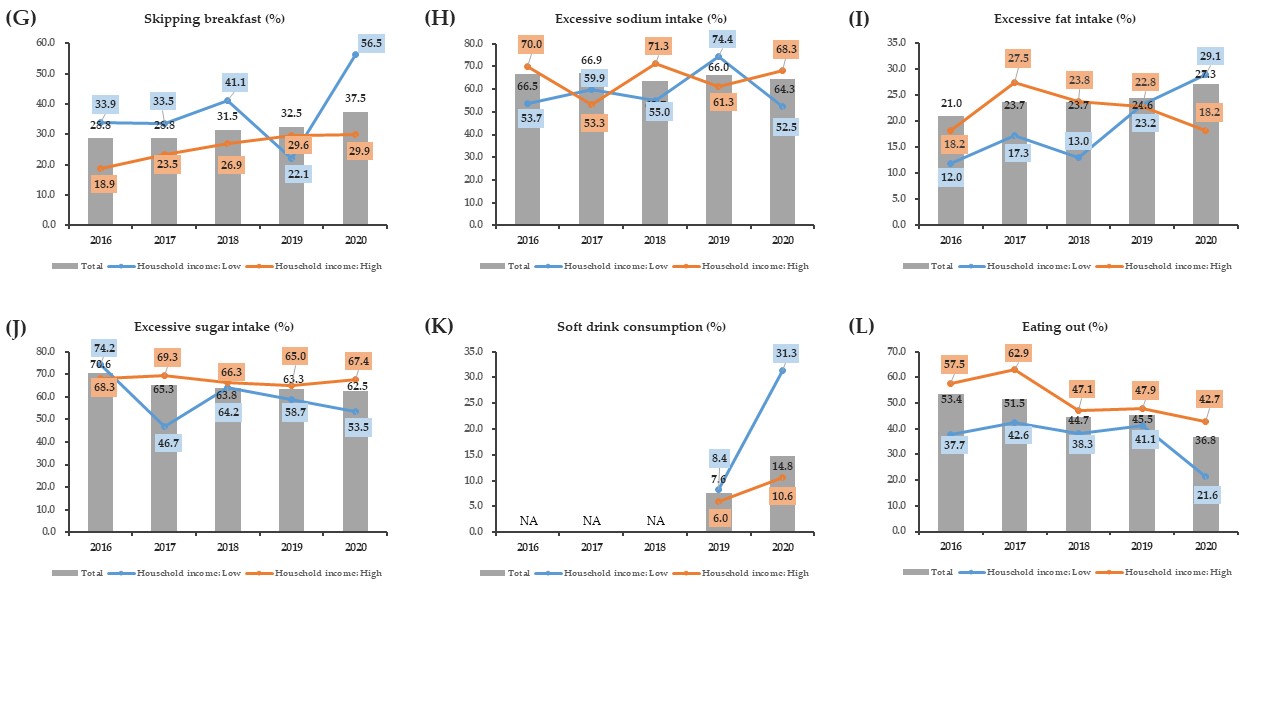

Supplement: Supplementary file 1 [file epih-44-e2022102-Supplementary-1.docx]
